# MRBrainS Challenge: Online Evaluation Framework for Brain Image Segmentation in 3T MRI Scans

**DOI:** 10.1155/2015/813696

**Published:** 2015-12-02

**Authors:** Adriënne M. Mendrik, Koen L. Vincken, Hugo J. Kuijf, Marcel Breeuwer, Willem H. Bouvy, Jeroen de Bresser, Amir Alansary, Marleen de Bruijne, Aaron Carass, Ayman El-Baz, Amod Jog, Ranveer Katyal, Ali R. Khan, Fedde van der Lijn, Qaiser Mahmood, Ryan Mukherjee, Annegreet van Opbroek, Sahil Paneri, Sérgio Pereira, Mikael Persson, Martin Rajchl, Duygu Sarikaya, Örjan Smedby, Carlos A. Silva, Henri A. Vrooman, Saurabh Vyas, Chunliang Wang, Liang Zhao, Geert Jan Biessels, Max A. Viergever

**Affiliations:** ^1^Image Sciences Institute, University Medical Center Utrecht, 3584 CX Utrecht, Netherlands; ^2^Philips Healthcare, 5680 DA Best, Netherlands; ^3^Faculty of Biomedical Engineering, Eindhoven University of Technology, 5600 MB Eindhoven, Netherlands; ^4^Department of Neurology, Brain Center Rudolf Magnus, University Medical Center Utrecht, 3584 CX Utrecht, Netherlands; ^5^Department of Radiology, University Medical Center Utrecht, 3584 CX Utrecht, Netherlands; ^6^BioImaging Laboratory, Bioengineering Department, University of Louisville, Louisville, KY 40292, USA; ^7^Biomedical Imaging Group Rotterdam, Departments of Medical Informatics and Radiology, Erasmus MC, 3015 CN Rotterdam, Netherlands; ^8^Department of Computer Science, University of Copenhagen, 2100 Copenhagen, Denmark; ^9^Image Analysis and Communications Laboratory, Department of Electrical and Computer Engineering, Johns Hopkins University, Baltimore, MD 21218, USA; ^10^Department of Electronics and Communication Engineering, The LNM Institute of Information Technology, Jaipur 302031, India; ^11^Imaging Laboratories, Robarts Research Institute, London, ON, Canada N6A 5B7; ^12^Department of Medical Biophysics, Western University, London, ON, Canada N6A 3K7; ^13^Signals and Systems, Chalmers University of Technology, 41296 Gothenburg, Sweden; ^14^Applied Physics Laboratory, Johns Hopkins University, Laurel, MD 20723, USA; ^15^Department of Electronics, University of Minho, 4800-058 Guimarães, Portugal; ^16^Department of Computing, Imperial College London, London SW7 2AZ, UK; ^17^Computer Science and Engineering Department, State University of New York at Buffalo, Buffalo, NY 14260-2500, USA; ^18^Center for Medical Imaging Science and Visualization, Linköping University, 58185 Linköping, Sweden; ^19^Department of Radiology and Department of Medical and Health Sciences, Linköping University, 58185 Linköping, Sweden

## Abstract

Many methods have been proposed for tissue segmentation in brain MRI scans. The multitude of methods proposed complicates the choice of one method above others. We have therefore established the MRBrainS online evaluation framework for evaluating (semi)automatic algorithms that segment gray matter (GM), white matter (WM), and cerebrospinal fluid (CSF) on 3T brain MRI scans of elderly subjects (65–80 y). Participants apply their algorithms to the provided data, after which their results are evaluated and ranked. Full manual segmentations of GM, WM, and CSF are available for all scans and used as the reference standard. Five datasets are provided for training and fifteen for testing. The evaluated methods are ranked based on their overall performance to segment GM, WM, and CSF and evaluated using three evaluation metrics (Dice, H95, and AVD) and the results are published on the MRBrainS13 website. We present the results of eleven segmentation algorithms that participated in the MRBrainS13 challenge workshop at MICCAI, where the framework was launched, and three commonly used freeware packages: FreeSurfer, FSL, and SPM. The MRBrainS evaluation framework provides an objective and direct comparison of all evaluated algorithms and can aid in selecting the best performing method for the segmentation goal at hand.

## 1. Introduction

Multiple large population studies [1–3] have shown the importance of quantifying brain structure volume, for example, to detect or predict small vessel disease and Alzheimer's disease. In clinical practice, brain volumetry can be of value in disease diagnosis, progression, and treatment monitoring of a wide range of neurologic conditions, such as Alzheimer's disease, dementia, focal epilepsy, Parkinsonism, and multiple sclerosis [4]. Automatic brain structure segmentation in MRI dates back to 1985 [5] and many methods have been proposed since then. However, the multitude of methods proposed [6–13] complicates the choice for a certain method above others. As early as 1986, Price [14] stressed the importance of comparing different approaches to the same type of problem. Various studies have addressed this issue and evaluated different brain structure segmentation methods [15–19]. However, several factors complicate direct comparison of different approaches. Not all algorithms are publicly available, and if they are, researchers who use them are generally not as experienced with these algorithms as they are with their own algorithm in terms of parameter tuning, which could result in a bias towards their own method. This problem does not exist when researchers apply their own method to publicly available data. Therefore, publicly available databases like the “Alzheimer's Disease Neuroimaging Initiative” (ADNI) (http://adni.loni.usc.edu/), the “Internet Brain Segmentation Repository” (IBSR) (http://www.nitrc.org/projects/ibsr), the CANDI Share Schizophrenia Bulletin 2008 (https://www.nitrc.org/projects/cs_schizbull08) [20], and Mindboggle (http://www.mindboggle.info/) [21] are important initiatives to enable comparison of various methods on the same data. However, due to the use of subsets of the available data and different evaluation measures, direct comparison can be problematic. To address this issue, grand challenges in biomedical image analysis were introduced in 2007 [22]. Participants in these competitions can apply their algorithms to the provided data, after which their results are evaluated and ranked by the organizers. Many challenges (http://grand-challenge.org/All_Challenges/) have been organized since then, providing an insight into the performance of automatic algorithms for specific tasks in medical image analysis.

In this paper we introduce the MRBrainS challenge evaluation framework (http://mrbrains13.isi.uu.nl/), an online framework to evaluate automatic and semiautomatic algorithms that segment gray matter (GM), white matter (WM), and cerebrospinal fluid (CSF) in 3T brain MRI scans of older (mean age 71) subjects with varying degrees of atrophy and white matter lesions. This framework has three main advantages. Firstly, researchers apply their own segmentation algorithms to the provided data. Parameters are optimally tuned to achieve the best possible performance. Secondly, all algorithms are applied to the exact same data and the reference standard of the test data is unknown to the participating researchers. Thirdly, the evaluation algorithm and measures are the same for all evaluated algorithms, enabling direct comparison of the various algorithms. The framework was launched at the MRBrainS13 challenge workshop at the Medical Image Computing and Computer Assisted Intervention (MICCAI) conference on September 26th in 2013. Eleven teams participated in the challenge workshop with a wide variety of segmentation algorithms, the results for which are presented in this paper and provide a benchmark for the proposed evaluation framework. In addition, we evaluated three commonly used freeware packages on the evaluation framework: FreeSurfer (http://surfer.nmr.mgh.harvard.edu/) [23, 24], FSL (http://fsl.fmrib.ox.ac.uk/fsl/fslwiki/) [25], and SPM (http://www.fil.ion.ucl.ac.uk/spm/) [26].

## 2. Materials and Methods

### 2.1. Evaluation Framework

The MRBrainS evaluation framework is set up as follows. Multisequence (T1-weighted, T1-weighted inversion recovery, and T2-weighted fluid attenuated inversion recovery) 3T MRI scans of twenty subjects are available for download on the MRBrainS website (http://mrbrains13.isi.uu.nl/). The data is described in more detail in Section 2.1.1. All scans were manually segmented into GM, WM, and CSF. These manual segmentations are used as the reference standard for the evaluation framework. The annotation process for obtaining the reference standard is described in Section 2.1.2. For five of the twenty datasets the reference standard is provided on the website and can be used for training an automatic segmentation algorithm. The remaining fifteen MRI datasets have to be segmented by the participating algorithms into GM, WM, and CSF. For these fifteen datasets, the reference standard is not provided online. The segmentation results can be submitted on the MRBrainS website. With each submission, a short description of the segmentation algorithm has to be provided, which should at least describe the algorithm, the used MRI sequences, whether the algorithm is semi- or fully automatic, and the average runtime of the algorithm. The segmentation results are then evaluated (Section 2.1.3) and ranked (Section 2.1.4) by the organizers and the results are presented on the website. More information on how to use the evaluation framework is provided in the details section of the MRBrainS website (http://mrbrains13.isi.uu.nl/details.php).

#### 2.1.1. Data

The focus was on brain segmentation in the context of ageing. Twenty subjects (mean age ± SD = 71 ± 4 years, 10 male, 10 female) were selected from an ongoing cohort study of older (65–80 years of age) functionally independent individuals without a history of invalidating stroke or other brain diseases [27]. This study was approved by the local ethics committee of the University Medical Center Utrecht (Netherlands) and all participants signed an informed consent form. To be able to test the robustness of the segmentation algorithms in the context of ageing-related pathology, the subjects were selected to have varying degrees of atrophy and white matter lesions. Scans with major artefacts were excluded. MRI scans were acquired on a 3.0 T Philips Achieva MR scanner at the University Medical Center Utrecht (Netherlands). The following sequences were acquired and used for the evaluation framework: 3D T1 (TR: 7.9 ms, TE: 4.5 ms), T1-IR (TR: 4416 ms, TE: 15 ms, and TI: 400 ms), and T2- FLAIR (TR: 11000 ms, TE: 125 ms, and TI: 2800 ms). Since the focus of the MRBrainS evaluation framework is on comparing different segmentation algorithms, we performed two preprocessing steps to limit the influence of different registration and bias correction algorithms on the segmentation results. The sequences were aligned by rigid registration using Elastix [28] and bias correction was performed using SPM8 [29]. After registration, the voxel size within all provided sequences (T1, T1 IR, and T2 FLAIR) was 0.96 × 0.96 × 3.00 mm^3^. The original 3D T1 sequence (voxel size: 1.0 × 1.0 × 1.0 mm^3^) was provided as well. Five datasets that were representative for the overall data (2 male, 3 female, varying degrees of atrophy and white matter lesions) were selected for training. The remaining fifteen datasets are provided as test data.

#### 2.1.2. Reference Standard

**Figure 1:**
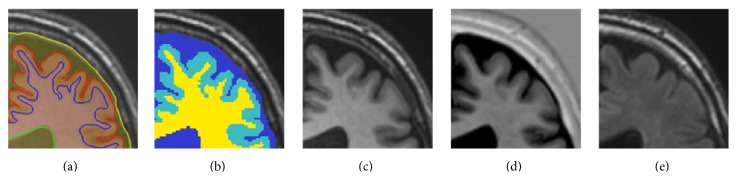
Example of the manually drawn contours (a), the resulting hard segmentation map (GM: light blue, WM: yellow, and CSF: dark blue) that is used as the reference standard (b), the T1-weighted scan (c), the T1-weighted inversion recovery (IR) scan (d), and the T2-weighted fluid attenuated inversion recovery (FLAIR) scan (e).

Manual segmentations were performed to obtain a reference standard for the evaluation framework. All axial slices of the 20 datasets (0.96 × 0.96 × 3.00 mm^3^) were manually segmented by trained research assistants in a darkened room with optimal viewing conditions. All segmentations were checked and corrected by three experts: a neurologist in training, a neuroradiologist in training, and a medical image processing scientist. To perform the manual segmentations, an in-house developed tool based on MeVisLab (MeVis Medical Solutions AG, Bremen, Germany) was used, employing a freehand spline drawing technique [30]. The closed freehand spline drawing technique was used to delineate the outline of each brain structure starting at the innermost structures (Figure 1(a)), working outward. The closed contours were converted to hard segmentations, and the inner structures were iteratively subtracted from the outer structures to construct the final hard segmentation image (Figure 1(b)). The following structures were segmented and are available for training: cortical gray matter (1), basal ganglia (2), white matter (3), white matter lesions (4), peripheral cerebrospinal fluid (5), lateral ventricles (6), cerebellum (7), and brainstem (8). These structures can be merged into gray matter (1, 2), white matter (3, 4), and cerebrospinal fluid (5, 6). The cerebellum and brainstem are excluded from the evaluation. All structures were segmented on the T1-weighted scans that were registered to the FLAIR scans, except for the white matter lesions (WMLs) and the CSF outer border (used to determine the intracranial volume). The WMLs were segmented on the FLAIR scan by the neurologist in training and checked and corrected by the neuroradiologist in training. The CSF outer border was segmented using both the T1-weighted and the T1-weighted IR scan, since the T1-weighted IR scan shows higher contrast at the borders of the intracranial volume. The CSF segmentation includes all vessels (including the superior sagittal sinus and the transverse sinuses) and nonbrain structures such as the cerebral falx and choroid plexuses.

#### 2.1.3. Evaluation

To evaluate the segmentation results we use three types of measures: a spatial overlap measure, a boundary distance measure, and a volumetric measure. The Dice [31] coefficient is used to determine the spatial overlap and is defined as

(1) $$\begin{array}{c} D=\frac{2\left|A\cap G\right|}{\left|A\right|+\left|G\right|}\cdot 100, \end{array}$$

where *A* is the segmentation result, *G* is the reference standard, and *D* is the Dice expressed as percentages. The 95th-percentile of the Hausdorff distance is used to determine the distance between the segmentation boundaries. The conventional Hausdorff distance uses the maximum, which is very sensitive to outliers. To correct for outliers, we use the 95th-percentile of the Hausdorff distance, by selecting the *K*th ranked distance as proposed by Huttenlocher et al. [32]:

(2) h95A,G=Ka∈Ath95ming∈G⁡g−a,

where ^95^ *K*_*a*∈*A*_^th^ is the *K*th ranked minimum Euclidean distance with *K*/*N*_*a*_ = 95%, *A* is the set of boundary points {*a*_1_,…, *a*_*N*_*a*__} of the segmentation result, and *G* is the set of boundary points {*g*_1_,…, *g*_*N*_*g*__} of the reference standard. The 95th-percentile of the Hausdorff distance is defined as

(3) $$\begin{array}{c} H_{95}\left(\;A,G\;\right)=\max\left(\;h_{95}\left(\;A,G\;\right),h_{95}\left(\;G,A\;\right)\;\right). \end{array}$$

The third measure is the percentage absolute volume difference, defined as

(4) $$\begin{array}{c} \text{AVD}=\frac{\left|V_{a}-V_{g}\right|}{V_{g}}\cdot 100, \end{array}$$

where *V*_*a*_ is the volume of the segmentation result and *V*_*g*_ is the volume of the reference standard. These measures are used to evaluate the following brain structures in each of the fifteen test datasets: GM, WM, CSF, brain (GM + WM), and intracranial volume (GM + WM + CSF). The brainstem and cerebellum are excluded from the evaluation.

#### 2.1.4. Ranking

To compare the segmentation algorithms that participate in the MRBrainS evaluation framework, the algorithms are ranked based on their overall performance to segment GM, WM, and CSF. Each of these components (*C* = {GM, WM, CSF}) is evaluated by using the three evaluation measures (*M* = {*D*, *H*_95_, AVD}) described in Section 2.1.3. For each component *c* ∈ *C* and each evaluation measure *m* ∈ *M*, the mean and standard deviation are determined over all 15 test datasets. The segmentation algorithms are then sorted on the mean *D* value in descending order and on the mean *H*_95_ and AVD value in ascending order. Each segmentation algorithm receives a rank (*r*) between 1 (ranked best) and *n* (number of participating algorithms) for each component *c* and each evaluation measure *m*. The final ranking is based on the overall score of each algorithm, which is the sum over all ranks, defined as

(5) $$\begin{array}{c} s=\overset{\left|M\right|}{\underset{m=0\;}{\sum }}\overset{\left|C\right|}{\underset{c=0}{\sum }}r_{mc}, \end{array}$$

where *r*_*mc*_ is the rank of the segmentation algorithm for measure *m* of component *c*. For the final ranking *r*, the overall scores *s* are sorted in ascending order and ranked from 1 to *n*. In case two or more algorithms have equal scores, the standard deviation over all 15 test datasets is taken into account to determine the final rank. The segmentation algorithms are then sorted on the standard deviation in ascending order and ranked for each component *c* and each evaluation measure *m*. The overall score is determined using (5) and the algorithms are sorted based on this score in ascending order and ranked from 1 to *n*. The algorithms that have equal overall scores based on the mean value are then ranked based on this standard deviation rank.

### 2.2. Evaluated Methods

The evaluation framework described in Section 2.1 was launched at the MRBrainS13 challenge workshop at the Medical Image Computing and Computer Assisted Intervention (MICCAI) conference on September 26th in 2013. For the workshop challenge, the test datasets were split into twelve off-site and three on-site test datasets. For the off-site part, teams could register on the MRBrainS website (http://mrbrains13.isi.uu.nl/) and download the five training and twelve test datasets. A time slot of eight weeks was available for teams to download the data, train their algorithms, segment the test datasets, and submit their results on the website. Fifty-eight teams downloaded the data, of which twelve submitted their segmentation results. The evaluation results were reported to the twelve teams and all teams submitted a workshop paper to the MRBrainS13 challenge workshop at MICCAI. Eleven teams presented their results at the workshop and segmented the three on-site test datasets live at the workshop within a time slot of 3.5 hours. These algorithms provide a benchmark for the proposed evaluation framework and are briefly described in Sections 2.2.1–2.2.11 in alphabetical order of teams' names. The teams' names are used in the paper to identify the methods. For a full description of the methods we refer to the workshop papers [33–43]. In Section 2.2.12 we describe the evaluated freeware packages.

#### 2.2.1. BIGR2 [37]

This multifeature SVM [37] method classifies voxels by using a Support Vector Machine (SVM) classifier [44] with a Gaussian kernel. Besides spatial features and intensity information from all three MRI sequences, the SVM classifier incorporates Gaussian-scale-space features to facilitate a smooth segmentation. Skull stripping is performed by nonrigid registration of the masks of the training images to the target image.

#### 2.2.2. Bigr_neuro [41]

This auto-kNN [45] method is based on an automatically trained kNN-classifier. First, a probabilistic tissue atlas is generated by nonrigidly registering the manually annotated atlases to the subject of interest. Training samples are obtained by thresholding the probabilistic atlas and subsequently pruning the feature space. White matter lesions are detected by applying an adaptive threshold, determined from the tissue segmentation, to the FLAIR sequence.

#### 2.2.3. CMIV [43]

A statistical-model-guided level-set method is used to segment the skull, brain ventricles, and basal ganglia. Then a skeleton-based model is created by extracting the midsurface of the gray matter and defining the thickness. This model is incorporated into a level-set framework to guide the cortical gray matter segmentation. The coherent propagation algorithm [46] is used to accelerate the level-set evolution.

#### 2.2.4. Jedi Mind Meld [42]

This method starts by preprocessing the data via anisotropic diffusion. For each 2D slice of a labeled dataset, the canny edge pixels are extracted, and the Tourist Walk is computed. This is done for axial, sagittal, and coronal views. Machine learning is used with these features to automatically label edge pixels in an unlabeled dataset. Finally, these labels are used by the Random Walker for automatic segmentation.

#### 2.2.5. LNMBrains [35]

The voxel intensities of all MRI sequences are modelled as a Gaussian distribution for each label. The parameters of the Gaussian distributions are evaluated as maximum likelihood estimates and the posterior probability of each label is determined by using Bayesian estimation. A feature set consisting of regional intensity, texture, spatial location of voxels, and the posterior probability estimates is used to classify each voxel into CSF, WM, GM, or background by using a multicategory SVM classifier.

#### 2.2.6. MNAB [38]

This method uses Random Decision Forests to classify the voxels into GM, WM, and CSF. It starts by a skull stripping procedure, followed by an intensity normalization of each MRI sequence. Feature extraction is then performed on the intensities, posterior probabilities, neighborhood statistics, tissue atlases, and gradient magnitude. After classification, isolated voxels are removed by postprocessing.

#### 2.2.7. Narsil [34]

This is a model-free algorithm that uses ensembles of decision trees [47] to learn the mapping from image features to the corresponding tissue label. The ensembles of decision trees are constructed from corresponding image patches of the provided T1 and FLAIR scans with manual segmentations. The N3 algorithm [48] was used for additional inhomogeneity correction and SPECTRE [49] was used for skull stripping.

#### 2.2.8. Robarts [39]

Multiatlas registration [50] with the T1 training images was used to propagate labels to generate sample histograms in a log-likelihood intensity model and probabilistic shape priors. These were employed in a MAP data term and regularized via computation of a hierarchical max-flow [51]. A brain mask from registration of the T1-IR training images was used to obtain the final results.

#### 2.2.9. S2_QM [36]

This method [52] is based on Bayesian-based adaptive mean shift and the voxel-weighted *K*-means algorithm. The former is used to segment the brain into a large number of clusters or modes. The latter is employed to assign these clusters to one of the three components: WM, GM, or CSF.

#### 2.2.10. UB VPML Med [40]

This method creates a multiatlas by registering the training images to the subject image and then propagating the corresponding labels to a fully connected graph on the subject image. Label fusion then combines the multiple labels into one label at each voxel with intensity similarity based weighted voting. Finally the method clusters the graph using multiway cut in order to achieve the final segmentation.

#### 2.2.11. UofL BioImaging [33]

This is an automated MAP-based method aimed at unsupervised segmentation of different brain tissues from T1-weighted MRI. It is based on the integration of a probabilistic shape prior, a first-order intensity model using a Linear Combination of Discrete Gaussians (LCDG), and a second-order appearance model. These three features are integrated into a two-level joint Markov-Gibbs Random Field (MGRF) model of T1-MR brain images. Skull stripping was performed using BET2 [40] followed by an adaptive threshold-based technique to restore the outer border of the CSF using both T1 and T1-IR; this technique was not described in [33], due to a US patent application [53], but is described in [54]. This method was applied semiautomatically to the MRBrainS test data, due to per scan parameter tuning.

#### 2.2.12. Freeware Packages

Next to the methods evaluated at the workshop, we evaluated three commonly used freeware packages for MR brain image segmentation: FreeSurfer (http://surfer.nmr.mgh.harvard.edu/) [23, 24], FSL (http://fsl.fmrib.ox.ac.uk/fsl/fslwiki/) [25], and SPM12 (http://www.fil.ion.ucl.ac.uk/spm/) [26]. All packages were applied using the default settings, unless mentioned otherwise. FreeSurfer (v5.3.0) was applied to the high resolution T1 sequence. The mri_label2vol tool was used to map the labels on the thick slice T1 that was used for the evaluation. FSL (v5.0) was directly applied to the thick slice T1 and provides both a pveseg and a seg file as binary output. We evaluated both of these files. The fractional intensity threshold parameter “*f*” of the BET tool that sets the brain/nonbrain intensity threshold was set according to [55] at 0.2 (Philips Achieva 3T setting). SPM12 was directly applied to the thick slice T1 sequence as well. However, it also provides the option to add multiple MRI sequences. Therefore we evaluated SPM12 not only on the thick slice T1 sequence but added the T1-IR and the T2-FLAIR scan as well and tested various combinations. The number of Gaussians was set according to the SPM manual to two for GM, two for WM, and two for CSF.

### 2.3. Statistical Analysis

All evaluated methods were compared to the reference standard. In summary of the results, the mean and standard deviation over all 15 test datasets were calculated per component (GM, WM, and CSF) and combination of components (brain, intracranial volume) and per evaluation measure (Dice, 95th-percentile Hausdorff distance, and absolute volume difference) for each of the evaluated methods. Boxplots were created using R version 3.0.3 (R project for statistical computing (http://www.r-project.org/)). Since white matter lesions should be segmented as white matter, the percentage of white matter lesion voxels segmented as white matter (sensitivity) was calculated for each algorithm over all 15 test datasets to evaluate the robustness of the segmentation algorithms against pathology.

## 3. Results

**Figure 2:**
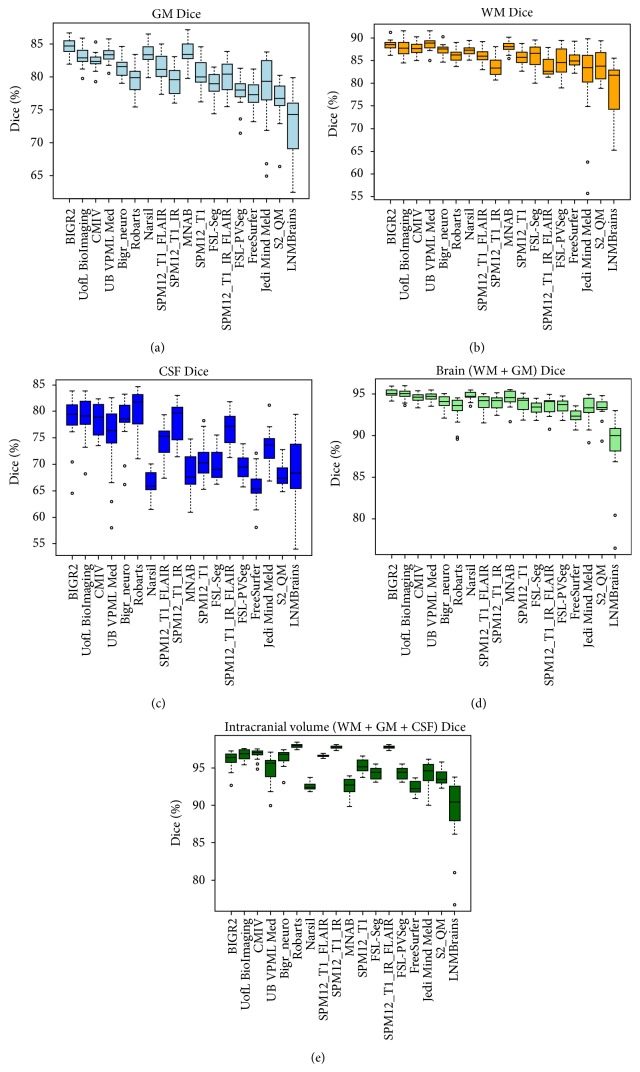
Boxplots presenting the evaluation results for the Dice coefficient (1) of the gray matter (GM), white matter (WM), cerebrospinal fluid (CSF), and brain and ICV segmentations for each of the participating algorithms and freeware packages over all 15 test datasets.

**Figure 3 fig3:**
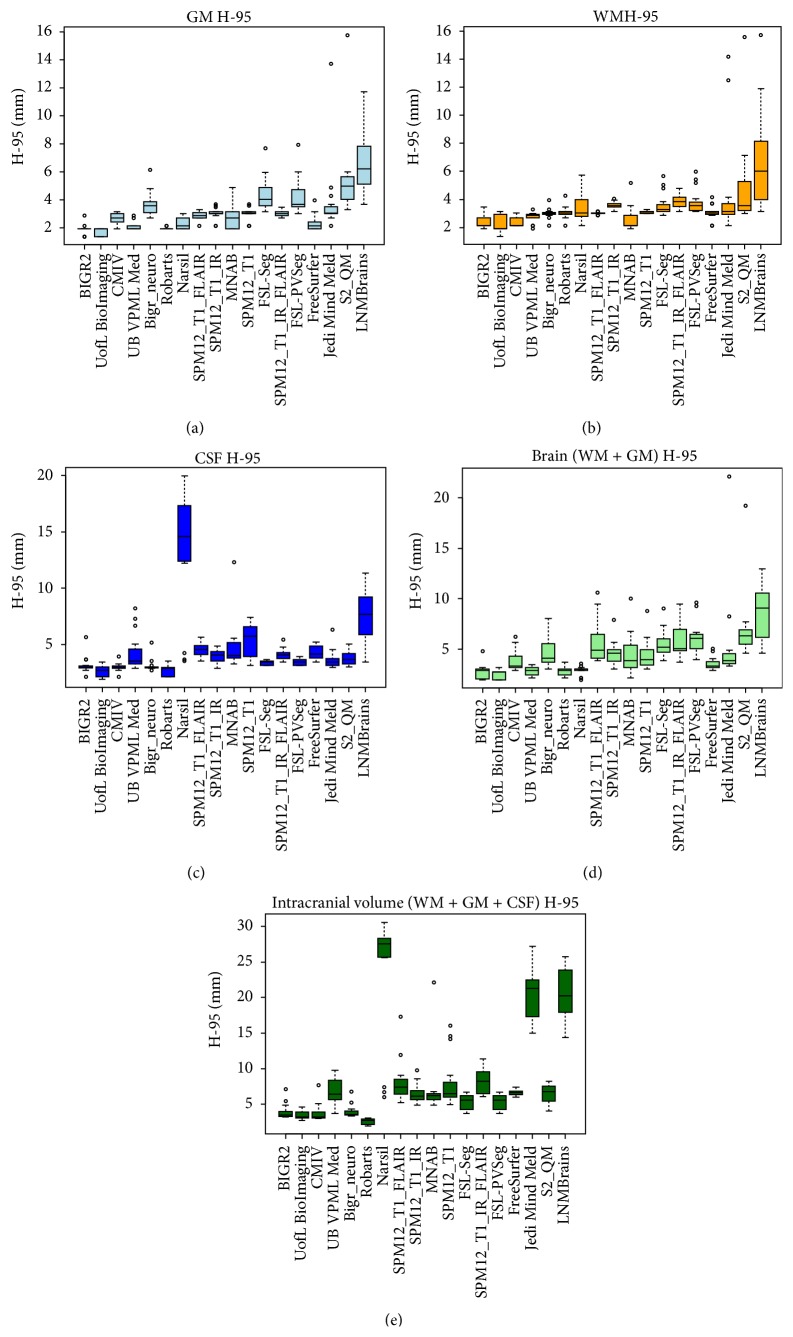
Boxplots presenting the evaluation results for the 95th-percentile Hausdorff distance (3) of the gray matter (GM), white matter (WM), cerebrospinal fluid (CSF), and brain and ICV segmentations for each of the participating algorithms and freeware packages over all 15 test datasets.

**Figure 4:**
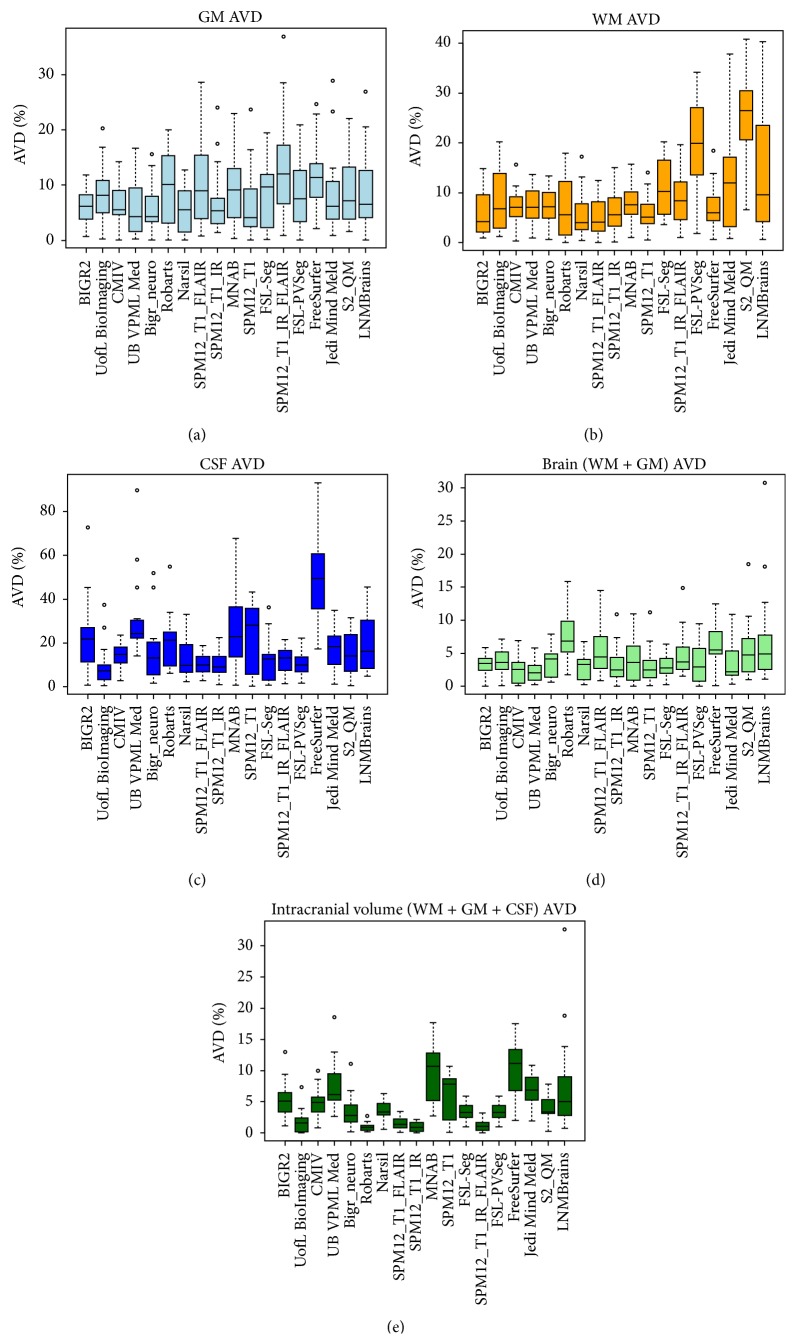
Boxplots presenting the evaluation results for the absolute volume difference (4) of the gray matter (GM), white matter (WM), cerebrospinal fluid (CSF), and brain and ICV segmentations for each of the participating algorithms and freeware packages over all 15 test datasets.

**Figure 5:**
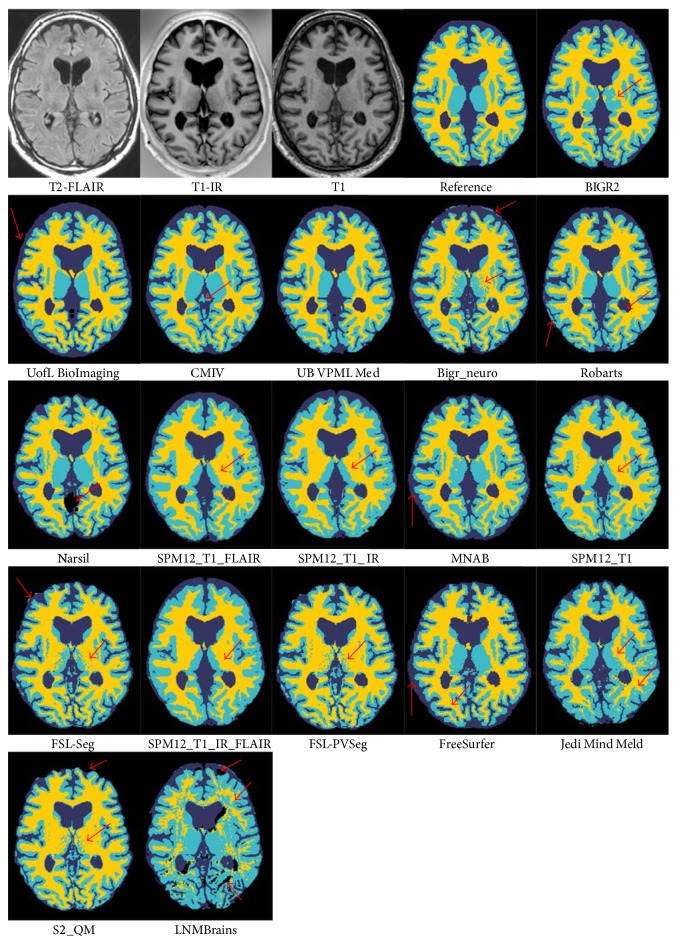
Illustration of the segmentation results at the height of the basal ganglia (test subject 9, slice 22). The basal ganglia should be segmented as gray matter. The first three images show the three MRI sequences. The fourth image (reference) is the manually segmented reference standard (yellow: white matter, light blue: gray matter, and dark blue: cerebrospinal fluid). The results of the participating segmentation algorithms are ordered from the best overall performance (BIGR2) to the worst overall performance (LNMBrains). Major differences are present in the segmentation of the basal ganglia and the outer border of the cerebrospinal fluid. The arrows indicate example locations where the segmentation results differ from the ground truth.

**Figure 6:**
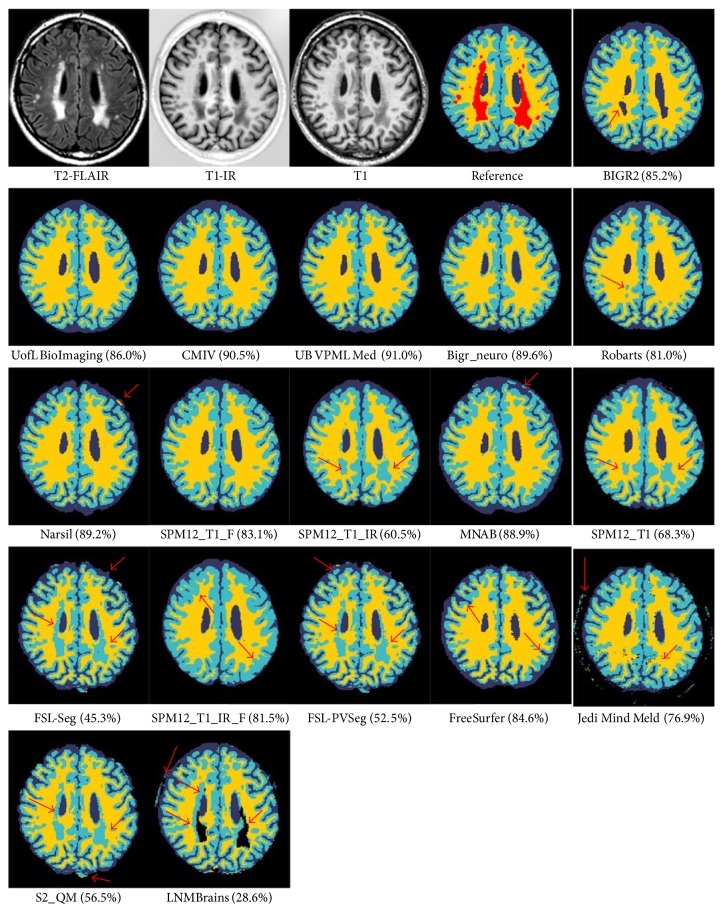
Illustration of the segmentation results in the presence of white matter lesions (test subject 3, slice 31). White matter lesions (WMLs) should be labeled as WM; the sensitivity (percentage of WML voxels is labeled as WM) over all 15 datasets is presented between brackets after the team name. The first three images show the three MRI sequences: T2-weighted fluid attenuated inversion recovery, T1-weighted inversion recovery, and T1-weighted scan. The fourth image (reference) is the manually segmented reference standard (red: white matter lesions, yellow: white matter, light blue: gray matter, and dark blue: cerebrospinal fluid). The results of the segmentation algorithms are ordered from the best overall performance (BIGR2) to the worst overall performance (LNMBrains). The arrows indicate example locations where the segmentation results differ from the ground truth.

Table 1 presents the final ranking (*r*) of the evaluated methods that participated in the workshop, as well as the evaluated freeware packages. During the workshop team UofL BioImaging ranked first and BIGR2 ranked second with one point difference in the overall score *s* (5). However, adding the results of the freeware packages resulted in an equal score for UofL BioImaging and BIGR2. Therefore the standard deviation rank was taken into account and BIGR2 is ranked first with standard deviation rank four and UofL BioImaging is ranked second with standard deviation rank eight. Table 1 further presents the mean, standard deviation, and rank for each evaluation measure (*D*, *H*_95_, and AVD) and component (GM, WM, and CSF), as well as the brain (WM + GM) and intracranial volume (WM + GM + CSF). Team BIGR2 scored best for the GM, WM, and brain segmentation and team UofL BioImaging for the CSF segmentation. Team Robarts scored best for the intracranial volume segmentation. The boxplots for all evaluation measures and components are shown in Figures 2 [Fig fig3]–4 and include the results of the freeware packages.  Figure 5 shows an example of the segmentation results at the height of the basal ganglia (slice 22 of test subject 9). The sensitivity of the algorithms to segment white matter lesions as WM and examples of the segmentation results in the presence of white matter lesions (slice 31 of test subject 3) are shown in Figure 6. Team UB VPML Med scores the highest sensitivity of white matter lesions segmented as white matter and is therefore most robust in the presence of this type of pathology.

## 4. Discussion

In this paper we proposed the MRBrainS challenge online evaluation framework to evaluate automatic and semiautomatic algorithms for segmenting GM, WM, and CSF on 3T multisequence (T1, T1-IR, and T2-FLAIR) MRI scans of the brain. We have evaluated and presented the results of eleven segmentation algorithms that provide a benchmark for algorithms that will use the online evaluation framework to evaluate their performance. Team UofL BioImaging and BIGR2 have equal overall scores, but BIGR2 was ranked first based on the standard deviation ranking. The evaluated methods represent a wide variety of algorithms that include Markov random field models, clustering approaches, deformable models, and atlas-based approaches and classifiers (SVM, KNN, and decision trees). The presented evaluation framework provides an insight into the performance of these algorithms in terms of accuracy and robustness. Various factors influence the choice for a certain method above others. We provide three measures that could aid in selecting the method that is most appropriate for the segmentation goal at hand: a boundary measure (95th-percentile Hausdorff distance *H*_95_), an overlap measure (Dice coefficient *D*), and a volume measure (absolute volume difference AVD). All three measures are taken into account for the final ranking of the methods. This ranking was designed to get a quick insight into how the methods perform in comparison to each other. The best overall method is the method that performs well for all three measures and all three components (GM, WM, and CSF). However, which method to select depends on the segmentation goal at hand. Not all measures are relevant for all segmentation goals. For example, if segmentation is used for brain volumetry [4], the overlap (*D*) and volume (AVD) measures of the brain and intracranial volume (used for normalization [56]) segmentations are important to take into account. On the other hand, if segmentation is used for cortical thickness measurements, the focus should be on the gray matter boundary (*H*_95_) and overlap (*D*) measures. Therefore the final ranking should be used to get a first insight into the overall performance, after which the performance of the measures and components that are most relevant for the segmentation goal at hand should be considered. Besides accuracy, robustness could also influence the choice for a certain method above others. For example, team UB VPML Med shows a high sensitivity score for segmenting white matter lesions as white matter (Figure 6) and shows a consistent segmentation performance of gray and white matter over all 15 test datasets (Figures 2–4). This could be beneficial for segmenting scans of populations with white matter lesions but is less important if the goal is to segment scans of young healthy subjects. In the latter case, the most accurate segmentation for gray and white matter (team BIGR2) is more interesting. If a segmentation algorithm is to be used in clinical practice, speed is an important consideration as well. The runtime of the evaluated methods is reported in Table 1. However, these runtimes are merely an indication of the required time, since academic software is generally not optimized for speed and the runtime is measured on different computers and platforms. Another relevant aspect of the evaluation framework is the comparison of multi- versus single-sequence approaches. For example, most methods struggle with the segmentation of the intracranial volume on the T1-weighted scan. There is no contrast between the CSF and the skull, and the contrast between the dura mater and the CSF is not always sufficient. Team Robarts used an atlas-based registration approach on the T1-IR scan (good contrast between skull and CSF) to segment the intracranial volume, which resulted in the best performance for intracranial volume segmentation (Table 1, Figures 2–4). Most methods add the T2-FLAIR scan to improve robustness against white matter lesions (Table 1, Figure 6). Although using only the T1-weighted scan and incorporating prior shape information (team UofL BioImaging) can be very effective also, the freeware packages support this as well. Since FreeSurfer is an atlas-based method, it uses prior information and is the most robust of all freeware packages to white matter lesions. However, adding the T2_FLAIR scan to SPM12 increases robustness against white matter lesions as well, as compared to applying SPM12 to the T1 scan only (Figure 6). In general SPM12 with the T1 and the T2-FLAIR sequence performs well in comparison to the other freeware packages (Table 1 and Figures 2–4) on the thick slice MRI scans. Although adding the T1-IR scan to SPM increases the performance of the CSF and ICV segmentations as compared to using only the T1 and T2-FLAIR sequence, it decreases the performance of the GM and WM segmentations. Therefore adding all sequences to SPM12 did not result in a better overall performance.

Besides the advantages of the MRBrainS evaluation framework, there are some limitations that should be taken into account. The T1-weighted IR and the T2-weighted FLAIR scan were acquired with a lower resolution (0.96 × 0.96 × 3.00 mm^3^) than the 3D T1-weighted scan (1.0 × 1.0 × 1.0 mm^3^). To be able to provide a registered multisequence dataset, the 3D T1-weighted scan was registered to the T2-weighted FLAIR scan and downsampled to 0.96 × 0.96 × 3.00 mm^3^. The reference standard is therefore only available for this resolution. The decreased performance of the FreeSurfer GM segmentation as compared to the other freeware packages might be due to the fact that we evaluate on the thick slice T1 sequence instead of the high resolution T1. Performing the manual segmentations to provide the reference standard is very laborsome and time consuming. Instead of letting multiple observers manually segment the MRI datasets or letting one observer manually segment the MRI datasets twice, much time and effort was spent on creating one reference standard that was as accurate as possible. Therefore we were not able to determine the inter- or intraobserver variability. Finally, we acknowledge that our evaluation framework is limited to evaluating the accuracy and robustness over 15 datasets for segmenting GM, WM, and CSF on 3T MRI scans acquired on a Philips scanner of a specific group of elderly subjects. Many factors influence segmentation algorithm performance, such as the type of scanner (vendor, field strength), the acquisition protocol, the available MRI sequences, and the type of subjects. Participating algorithms might have been designed for different types of MRI scans. Therefore the five provided training datasets are important for participants to be able to train their algorithms on the provided data. Some algorithms are designed to segment only some components, such as only GM and WM, instead of all three components, and use freely available software such as the brain extraction tool [57] to segment the outer border of the CSF (intracranial volume). We have chosen to base the final ranking on all three components, but it is therefore important to assess not only the final ranking, but the performance of the individual components as well.

Despite these limitations, the MRBrainS evaluation framework provides an objective and direct comparison of segmentation algorithms. The reference standard of the test data is unknown to the participants, the same evaluation measures are used for all evaluated algorithms, and participants apply their own algorithms to the provided data.

In comparison to the online validation engine proposed by Shattuck et al. [58], the MRBrainS evaluation framework uses 3T MRI data instead of 1.5T MRI data and evaluates not only brain versus nonbrain segmentation, but also segmentation of gray matter, white matter, cerebrospinal fluid, brain, and intracranial volume. The availability of many different types of evaluation frameworks will aid in the development of more generic and robust algorithms. For example, in the NEATBrainS (http://neatbrains15.isi.uu.nl/) challenge, researchers were challenged to apply their algorithms to data from both the MRBrainS and the NeoBrainS [59] (brain tissue segmentation in neonates) challenge. Two methods [60–62] specifically designed for neonatal brain tissue segmentation showed a high performance for tissue segmentation on the MRBrainS data. Applying algorithms to different types of data has the potential to lead to new insights and more robust algorithms. The MRBrainS evaluation framework remains open for new contributions. At the time of writing, 21 teams had submitted their results on the MRBrainS website (http://mrbrains13.isi.uu.nl/results.php).

## 5. Conclusion

The MRBrainS challenge online evaluation framework provides direct and objective comparison of automatic and semiautomatic methods to segment GM, WM, CSF, brain, and ICV on 3T multisequence MRI data. The first eleven participating methods are evaluated and presented in this paper, as well as three commonly used freeware packages (FreeSurfer, FSL, and SPM12). They provide a benchmark for future contributions to the framework. The final ranking provides a quick insight into the overall performance of the evaluated methods in comparison to each other, whereas the individual evaluation measures (Dice, 95th-percentile Hausdorff distance, and absolute volume difference) per component (GM, WM, CSF, brain, and ICV) can aid in selecting the best method for a specific segmentation goal.

## Figures and Tables

**Table 1:** Results of the 11 evaluated algorithms presented at the workshop and the evaluated freeware packages on the 15 test datasets. The algorithms are ranked (*r*) based on their overall score (*s*) by using (5). This score is based on the ranks of the gray matter (GM), white matter (WM), and cerebrospinal fluid (CSF) segmentation and the three evaluated measures: Dice coefficient (*D* in %), 95th-percentile Hausdorff distance (*H*_95_ in mm), and the absolute volume difference (AVD in %). The rank *r*_*mc*_ denotes the rank based on the mean (*μ*) over all 15 test datasets for each measure *m* (0: *D*, 1: *H*_95_, and 2: AVD) and component *c* (0: GM, 1: WM, 2: CSF, 3: brain (WM + GM), and 4: intracranial volume (ICV = WM + GM + CSF)). Teams BIGR2 and UofL BioImaging, and FreeSurfer and Jedi Mind Meld have equal scores based on the mean (*μ*); therefore the ranking based on the standard deviation (*σ*) is taken into account to determine the final rank (BIGR2: *σ* rank 4, UofL BioImaging: *σ* rank 8, FreeSurfer: *σ* rank 13, and Jedi Mind Meld: *σ* rank 17). Columns 2 and 3 present the average runtime *t* per scan in seconds (s), minutes (m), or hours (h) and the scans (T1: T1-weighted scan, 3D T1: 3D T1-weighted scan, IR: T1-weighted inversion recovery (IR), and F: T1-weighted FLAIR) that are used for processing.

*r*	Team	*t*	Scans	GM			WM			CSF			*s*	Brain			ICV		
				*r*_00_	*r*_10_	*r*_20_	*r*_01_	*r*_11_	*r*_21_	*r*_02_	*r*_12_	*r*_22_		*r*_03_	*r*_13_	*r*_23_	*r*_04_	*r*_14_	*r*_24_
				*D*	*H*_95_	AVD	*D*	*H*_95_	AVD	*D*	*H*_95_	AVD		*D*	*H*_95_	AVD	*D*	*H*_95_	AVD
				*μ* (*σ*)	*μ* (*σ*)	*μ* (*σ*)	*μ* (*σ*)	*μ* (*σ*)	*μ* (*σ*)	*μ* (*σ*)	*μ* (*σ*)	*μ* (*σ*)		*μ* (*σ*)	*μ* (*σ*)	*μ* (*σ*)	*μ* (*σ*)	*μ* (*σ*)	*μ* (*σ*)
1	BIGR2	35 m	T1, IR, F	1	2	4	2	2	4	4	5	14	38	1	2	12	8	4	12
				84.7	1.9	6.1	88.4	2.4	6.0	78.3	3.2	23		95.1	2.7	3.2	96.0	3.9	5.2
				(1.3)	(0.4)	(3.3)	(1.2)	(0.5)	(5.1)	(5.0)	(0.8)	(17)		(0.5)	(0.8)	(1.6)	(1.3)	(1.1)	(3.0)

2	UofL BioImaging^*∗*^	6 s	T1	5	1	9	4	1	13	2	2	1	38	2	1	12	5	2	5
				83.0	1.7	8.6	87.9	2.2	8.7	78.9	2.7	9.7		94.9	2.4	3.9	96.7	3.4	1.8
				(1.5)	(0.3)	(5.4)	(2.0)	(0.6)	(6.6)	(4.2)	(0.5)	(10)		(0.6)	(0.5)	(2.0)	(0.8)	(0.6)	(2.0)

3	CMIV	3 m	T1, F	6	7	5	5	3	10	3	3	8	50	5	7	2	4	3	11
				82.4	2.7	6.8	87.7	2.4	7.3	78.6	3.0	14		94.5	3.8	2.6	96.8	3.8	4.9
				(1.4)	(0.4)	(4.0)	(1.6)	(0.4)	(3.8)	(3.1)	(0.4)	(5.9)		(0.5)	(1.1)	(2.2)	(0.8)	(1.3)	(2.3)

4	UB VPML Med	30 m	T1, IR, F	4	4	2	1	5	7	8	13	17	61	4	4	1	10	11	15
				83.3	2.1	5.9	88.6	2.7	7.1	74.8	4.3	31		94.6	2.8	2.4	94.8	6.6	7.7
				(1.3)	(0.3)	(5.3)	(1.7)	(0.4)	(3.8)	(7.1)	(1.7)	(19)		(0.6)	(0.4)	(1.8)	(2.0)	(2.0)	(4.1)

5	Bigr_neuro	2 h	T1, F	7	13	3	6	6	9	6	4	10	64	7	10	10	7	5	8
				81.5	3.7	5.9	87.3	3.0	7.3	78.2	3.2	16		94.0	4.6	3.6	96.3	3.9	3.5
				(1.7)	(0.9)	(4.2)	(1.4)	(0.4)	(3.8)	(4.7)	(0.6)	(14)		(0.8)	(1.4)	(2.4)	(1.2)	(0.9)	(2.7)

6	Robarts	16 m	3D T1, IR	11	3	15	8	8	6	1	1	13	66	16	3	18	1	1	1
				79.7	2.0	9.8	86.2	3.1	7.1	80.3	2.7	20		93.1	2.8	7.9	97.9	2.6	0.9
				(2.4)	(0.1)	(7.3)	(1.3)	(0.4)	(6.2)	(4.1)	(0.5)	(13)		(1.6)	(0.5)	(3.6)	(0.3)	(0.4)	(0.7)

7	Narsil	2 m	T1, F	3	5	1	7	11	2	17	18	7	71	3	5	3	16	18	9
				83.5	2.3	5.5	87.1	3.3	5.8	66.6	13.3	14		94.8	2.9	2.9	92.5	24	3.7
				(1.8)	(0.4)	(4.4)	(1.3)	(0.9)	(5.3)	(2.4)	(5.4)	(9.5)		(0.5)	(0.5)	(2.0)	(0.5)	(8.9)	(1.7)

8	SPM_T1_F	3 m	T1, F	8	9	16	9	7	1	9	14	2	75	9	14	14	6	14	4
				81.2	2.9	10	86.0	3.0	5.2	74.1	4.6	10		93.9	5.8	5.3	96.6	8.2	1.5
				(2.2)	(0.3)	(8.5)	(1.5)	(0.1)	(3.8)	(3.4)	(0.6)	(4.7)		(1.0)	(2.2)	(3.8)	(0.2)	(3.0)	(1.0)

9	SPM_T1_IR	3 m	T1, IR	12	11	7	16	12	5	5	10	3	81	8	11	7	2	8	2
				79.4	3.0	7.2	83.5	3.6	6.3	78.3	4.0	10		93.9	4.6	3.4	97.7	6.5	1.0
				(2.1)	(0.4)	(6.3)	(2.1)	(0.3)	(4.6)	(3.8)	(0.6)	(5.7)		(0.8)	(1.2)	(2.8)	(0.2)	(1.3)	(0.8)

10	MNAB	15 m	T1, IR, F	2	8	12	3	4	11	15	15	16	86	6	9	11	15	12	17
				83.9	2.8	9.1	88.0	2.7	7.8	68.1	4.9	29		94.5	4.5	3.8	92.5	7.1	9.7
				(2.1)	(0.9)	(6.5)	(1.2)	(0.8)	(4.0)	(4.0)	(2.2)	(21)		(1.0)	(2.0)	(3.2)	(1.1)	(4.2)	(4.7)

11	SPM_T1	3 m	T1	9	10	6	11	10	3	11	16	15	91	10	8	6	9	13	13
				80.3	3.0	6.9	85.6	3.1	6.0	70.7	5.3	23		93.9	4.4	3.2	95.3	8.1	5.5
				(2.4)	(0.5)	(6.8)	(1.7)	(0.1)	(4.1)	(3.8)	(1.5)	(15.7)		(0.9)	(1.6)	(2.9)	(0.9)	(3.7)	(3.7)

12	FSL_Seg	10 m	T1	13	16	10	10	13	14	12	6	5	99	13	13	4	12	6	6
				78.7	4.3	8.6	86.0	3.7	11.5	69.9	3.4	12		93.3	5.5	3.0	94.2	5.3	3.4
				(2.2)	(1.2)	(6.3)	(2.6)	(0.8)	(6.3)	(2.8)	(0.2)	(10.3)		(0.8)	(1.4)	(1.5)	(0.8)	(1.1)	(1.5)

13	SPM_T1_IR_F	4 m	T1, IR, F	10	12	18	15	14	12	7	11	6	105	12	15	13	3	15	3
				80.1	3.0	13.9	83.6	3.8	8.4	76.9	4.1	12		93.6	5.9	5.1	97.7	8.2	1.2
				(2.4)	(0.2)	(9.6)	(2.1)	(0.5)	(5.2)	(3.1)	(0.5)	(6.0)		(1.1)	(1.8)	(3.6)	(0.2)	(1.8)	(0.9)

14	FSL _PVSeg	10 m	T1	15	15	8	13	15	17	13	7	4	107	11	16	9	13	7	7
				77.7	4.3	8.4	84.8	3.8	19.7	69.5	3.4	11		93.6	6.1	3.5	94.2	5.3	3.4
				(2.6)	(1.3)	(6.5)	(3.2)	(0.9)	(10)	(2.2)	(0.3)	(5.8)		(0.9)	(1.6)	(3.0)	(0.8)	(1.1)	(1.5)

15	FreeSurfer	1 h	3D T1	16	6	17	12	9	8	18	12	18	116	17	6	16	17	10	18
				77.4	2.3	12.1	85.2	3.1	7.2	65.8	4.3	50		92.3	3.6	6.3	92.4	6.6	10
				(2.0)	(0.6)	(6.0)	(2.2)	(0.5)	(4.6)	(3.7)	(0.6)	(19.6)		(0.8)	(0.6)	(3.4)	(0.9)	(0.4)	(4.5)

16	Jedi Mind Meld	27 s	T1, IR, F	14	14	11	17	16	15	10	8	11	116	15	12	8	11	16	14
				77.8	3.9	8.9	80.6	4.5	13.7	73.3	3.7	18		93.2	5.4	3.5	94.3	20	6.8
				(5.9)	(2.8)	(7.6)	(9.6)	(3.6)	(12)	(3.5)	(0.8)	(9.0)		(1.7)	(4.8)	(3.0)	(1.7)	(3.7)	(3.1)

17	S2_QM	1.5 h	T1, IR, F	17	17	13	14	17	18	16	9	9	130	14	17	15	14	9	10
				76.4	5.5	9.3	83.9	4.9	24.8	67.9	3.8	14		93.3	7.0	5.5	93.7	6.5	4.0
				(3.4)	(3.0)	(6.4)	(3.4)	(3.2)	(10)	(2.3)	(0.6)	(9.9)		(1.4)	(3.5)	(4.5)	(1.1)	(1.4)	(2.2)

18	LNMBrains	5 m	T1, IR, F	18	18	14	18	18	16	14	17	12	145	18	18	17	18	17	16
				72.8	6.8	9.5	78.3	6.8	15.3	68.8	7.7	20		88.5	8.6	7.4	89.2	20.4	7.9
				(5.3)	(2.3)	(7.6)	(6.4)	(3.5)	(13)	(6.6)	(2.4)	(13)		(4.5)	(2.8)	(7.7)	(4.8)	(3.7)	(8.3)

^*∗*^Semiautomatic due to per scan parameter tuning.
